# Supplementary material for: Antiviral Effects of Houttuynia cordata Polysaccharide Extract on Murine Norovirus-1 (MNV-1)—A Human Norovirus Surrogate
Source: Molecules. 2019 May 13;24(9):1835. doi: 10.3390/molecules24091835 (PMC6539669; doi:10.3390/molecules24091835)
Supplement: Supplementary file 1 [file molecules-24-01835-s001.pdf]

Antiviral Effects of *Houttuynia cordata* Polysaccharide Extract on Murine Norovirus-1 (MNV-1)—A Human Norovirus Surrogate

Dongqing Cheng [1,2,†], Liang Sun[1,†], Songyan Zou [2], Jiang Chen [1], Haiyan Mao [1], Yanjun Zhang [1] , Ningbo Liao [1,3,*] and Ronghua Zhang [1,2,*]

1 Department of Nutrition and Food Safety, Zhejiang Provincial Center for Disease Control and Prevention, Hangzhou 310006, China; chengdq@zcmu.edu.cn (D.C.); lsun@cdc.zj.cn (L.S.); jchen@cdc.zj.cn (J.C.); hymao@cdc.zj.cn (H.M.); yjzhang@cdc.zj.cn (Y.Z.)

2 College of Medical Technology, Zhejiang Chinese Medical University, Hangzhou 310053, China; zou_songyan@126.com

3 School of Public Health, Division of Infectious Diseases and Vaccinology, University of California, Berkeley, California 94720, USA

* Correspondence: liaoningbo2010@126.com (N.L.); rhzhang@cdc.zj.cn (R.Z.); Tel./Fax: +86-571-87115140 (N.L.); Tel./Fax: +86-571-87115214 (R.Z.)

† These authors contributed equally to this work.

Supporting information

Table S1. ^{1}H-NMR (D_2O, 500MHz) and ^{13}C-NMR (D_2O, 125MHz) spectra data of HP.

Residue	^{1}H and ^{13}C-NMR data(D_2O)of HP (δ in ppm)			
	Carbon	δ_C (ppm)	Proton	δ_H (ppm)
α-D-GalA (1→4)	C-1	99.3	H-1	5.09
	C-2	70.2	H-2	3.76
	C-3	71.4	H-3	3.92
	C-4	80.5	H-4	4.38
	C-5	73.4	H-5	4.68 [a]
	C-6	176.4	H-6	
β-D-Xyl (1→4)	C-1	103.1	H-1	4.65 [a]
β-D-Gal (1→4)	C-1	105.5	H-1	4.67 [a]
β-D-Glc (1→4)	C-1	104.4	H-1	4.62 [a]

[a] Overlapped with H_2O.

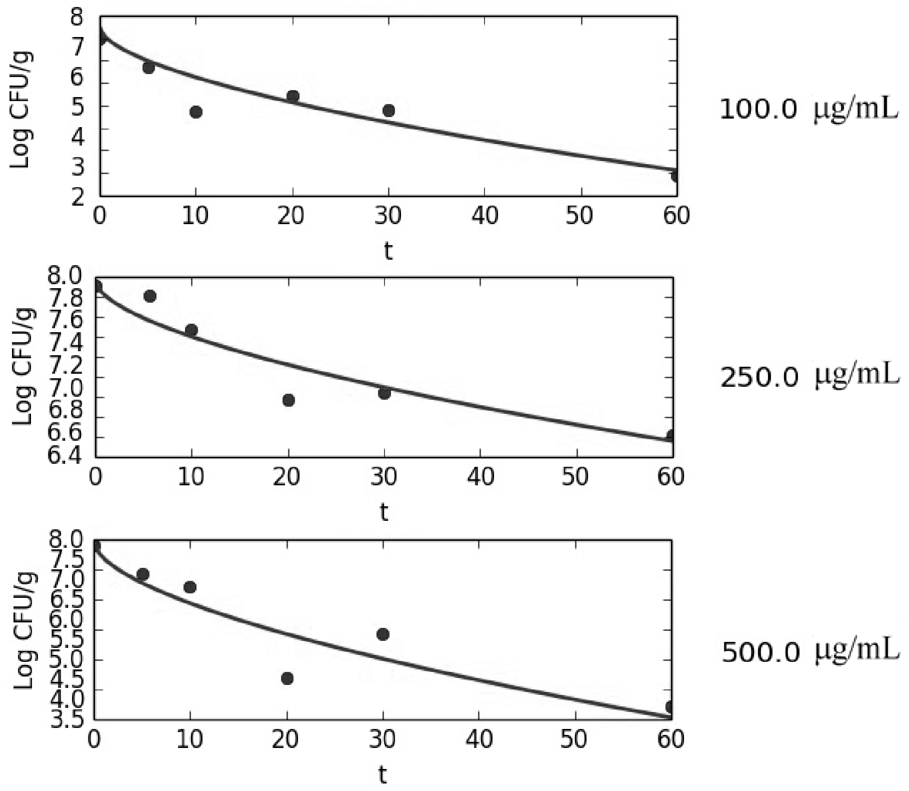

Figure S1. Weibull model for MNV-1 inactivation by HP under long-term incubation. MNV-1 was mixed with 100, 250 and 500 mg/mL of HP and incubated for 5, 10, 20, 30 and 60 min, respectively, at room temperature.
